# Iranian nursing students experiences regarding the status of e-learning during COVID-19 pandemic

**DOI:** 10.1371/journal.pone.0263388

**Published:** 2022-02-02

**Authors:** Naiire Salmani, Imane Bagheri, Atena Dadgari

**Affiliations:** 1 Nursing Faculty, Meybod Nursing School, Shahid Sadoughi University of Medical Sciences, Yazd, Iran; 2 College of Nursing and Midwifery, Isfahan University of Medical Sciences, Isfahan, Iran; Satyawati College (Eve.), University of Delhi, INDIA

## Abstract

**Introduction:**

With the emergence of the COVID-19 pandemic, universities immediately responded to protect students’ lives by implementing e-learning in order to stop the spread of the communicable disease within the academic population. This study aimed to describe iranian nursing students’ experiences of e-learning during the COVID-19 pandemic.

**Materials and methods:**

The current study used a qualitative descriptive study. Ten nursing undergraduate students from a single Iranian university identified using purposive sampling methods. Face-to-face semi-structured interview conducted from May to July 2021 and analyzed through thematic analysis. Lincoln and Goba criteria were used to obtain data validity and reliability.

**Results:**

Four themes emerged including"novelty of e-learning","advantages of e-learning", "disadvantages of e-learning"and"passage of time and the desire to return to face education". Participants evaluated e-learning as a novel method without proper infrastructure, it was initially confusing but became the new normal as their knowledge of the way to use it improved. Advantages included self-centered flexible learning and reduction in their concerns experienced with face-to-face learning. Disadvantages including changing the way they interact with teachers, decreasing interactions with classmates, problems with education files, superficial learning, hardware problems, family members’ perceptions of the student role, interference of home affairs with e-learning, cheating on exams and assignments and being far away from the clinical context.

**Conclusion:**

The findings revealed that e-learning has been introduced as a new method for the current research participants and despite the perceived benefits, these students believed that e-learning could supplement face education but not replace it.

## Introduction

Coronavirus 2019 (COVID-19, SARS-CoV-2) is the latest infectious disease that has quickly spread throughout the world [1] becoming a critical global epidemic [2]. Accordingly, universities around the world immediately responded by suspending regular teaching in classrooms and rapidly shifting to online education in order to protect students’ lives and stop the spread of this communicable disease [3]. The development of e-learning was originally aimed to increase access for students and compensate for deficiencies in in-person, face-to-face education [4]. However, in rapidly changing the prevailing modality of education from in-person face-to-face learning to E-learning [5], there has been significant concern about the quality of educational content and resources as nursing students lost direct face-to-face exposure to their two primary modalities of learning: face-to-face interaction with their teachers and fellow students, and clinical practice with their patients [6].

As the COVID-19 pandemic emerged, nursing professors were required to rapidly assess and decide on educational solutions for nursing students to continue their education; all of these solutions involved rapid deployment of E-learning to replace traditional face- education [7]. With fears that the social isolation caused by the COVID-19 outbreak would last for months, there were concerns about how to compensate for missed didactic and practical lessons as well as clinical experiences that are course requirements for student learning, creating concern about not being ready enough to enter the professional internship course in their final semesters of the undergraduate nursing program [8] so, it is essential for educators to understand students ’perceptions of E-learning and, students’ readiness to learn in this new environment. This perception can increase student attendance, satisfaction, and motivate students, and ultimately provide a meaningful and effective learning environment [9].

Also this issue is more important for nursing students because most of the education in the field of nursing is physical and the compulsion due to the spread of the disease to use E-learning can be associated with problems for students and teachers [10] because under the circumstances, the ability of nursing students to receive safe education in hospitals was disrupted and the demand for distance education increased. In order to use this method effectively in nursing, it is necessary to develop instructions. Therefore, it is necessary to explain students’ perceptions of this method, benefits and challenges of implementing in education [11].

Several studies have been conducted to examine health profession students’ perspectives on e-learning within the context of the prevailing situational changes in student education during the COVID-19 disease pandemic. Among Nigerian nursing students, online and virtual learning methods were considered user-friendly and it was easy to be skillful but there were barriers such as lack of regular internet access, low student participation and a lack of group leadership [12].Nearly one-third (30%) of Saudi Arabian pharmacy students stated they were required to be educated about online courses but considered that online learning has technical barriers [13]. Studies with medical students conducted during the COVID 19 outbreak have found that few prefer e-learning as the only learning modality [14], but preferences for combined online and traditional learning were expressed [15]. Online learning has certain advantages including time savings on travel, flexibility with scheduling and student learning speed, and cost [5]. Lack of direct contact with professors was the most significant disadvantage [15], as well as logistical constraints such as space, being on time for synchronous classes, and family distractions [5].

In Iran, nursing and medical students are educated in face-to-face format. Prior to the outbreak of Covid-19 disease, virtual education did not have a defined position in teaching in the health sciences, Virtual education began with the sudden closure of universities; the Deputy Minister of Education of the Ministry of Health and Medical Education of Iran introduced a system called Navid with Adobe Connect software supporting for virtual education in online and offline formats. By activating the camera and microphone, it was possible for professors to hold online classes where the students could see and talk to the teacher while teaching. In offline education, teachers prepared the educational files in various forms according to the professors’ preferences, including voice and/or video of the content, and the slides in Power point, which were uploaded in the Navid system. At Shahid Sadoughi University of Medical Sciences in Yazd where the study was conducted, the Navid system and Adobe Connect software were installed but no training courses were held for teachers and students to use this system. Internet connectivity was provided without charge to professors and students.

While prior studies have concentrated on the student perceptions of the differences in traditional and E-learning methods, examining and acknowledging students perceptions and expectations of their experience of changing educational methods during the outbreak of COVID-19 can provide insights for educational managers to enable them to identify the advantages and disadvantages of current educational methods and assist improvement in the delivery of education for the current and future pandemic crises [5]. Consequently, this study was conducted in order to explain the iranian nursing students’ experiences with e-learning during the COVID-19 pandemic.

## Materials and methods

### Design

This study used a qualitative design conducted with in-depth individual interviews, to describe the perceptions of Iranian nursing students within their reality of the COVID-19 situation. The ethics committee of Yazd University of Medical Sciences approved the study.

### Sample and data collection

Using purposive sampling, nursing students who had successfully completed the 4^th^, 6^th^ and 8^th^ semester of the undergraduate nursing program were recruited to the study. An announcement was posted on the school website stating the title of the study and its purpose. Students willing to volunteer were asked to contact the researchers for more information about participation. The researchers’ telephone numbers were provided in the announcement.

A semi-structured interview was conducted which involved several open-ended questions that were designed by the research team. Questions included:

"What do you understand when you hear the term E- education?"

" How do you understand your role as a student in E-learning education?"

"Describe your experience with the implementation of E-Learning during this period?"

The interviews were conducted in the researcher’s work office. Participants received full oral explanations about the research objectives and methods. Before the interviews were conducted, the researchers received verbal and written consent, including permission to audio-record the interview. Participants were reassured that their participation was completely voluntary, they were allowed to leave the study at any time, and all audio-recordings would be kept confidential. The interviews lasted 20 to 45 minutes. The researchers are also faculty professors, hence, any identifying information was eliminated during the transcription of interviews before analyzing data.

### Data analysis

We used inductive content analysis to analyze data applying Graneheim and Lundma method. The data were qualitatively analyzed in five stages, including 1) transcribing the entire interview immediately after completion, 2) reading the entire text to gain a general understanding / summary of the content, 3) determining the units of meaning and basic codes, 4) Categorize similar primary codes into more comprehensive categories, and 5) determine the main theme of the categories [16]. After each interview, two researchers analyzed the interview transcripts, identifying specific semantic units, and coding line-by-line so that key phrases were extracted from the text and transferred to codes without changing the original content. Codes were then classified into themes and subcategories based on content similarity. The research team agreed on the codes, themes, and subcategories.

### Rigor

Lincoln and Goba criteria were used to obtain data validity and reliability [17]. To ensure validity, the researcher was relevant to the subject, data, and participants for 6 months, from January 2021 to June 2021. After coding, the check and review of the codes was used by the research team. Emerging categories and subcategories were also shared with participants as they progressed. For validity, transcripts were returned to participants after coding for validation of the extracted codes. Finally, the research team tried to have the maximum variety in sampling.

## Results

Ten nursing students from the 4^th^, 6^th^ and 8^th^ semesters participated in this study. There were 6 female students and 4 male students. Data analysis identified four themes with sub-categories. Table 1 presents examples of participant quotes for each theme and subcategory.

**Table 1:** Theme and subcategory.

Theme
Theme1. Novelty of e-learning method
a. Weakness of infrastructure
b. Student unfamiliarity
Theme 2: Advantages of virtual education
Flexible self-centered learning
Reducing concerns with face education
Theme 3: Disadvantages of e-learning
Poor communication between professors and students
Decreasing the interactions among the classmates
Problems with education files
Superficial learning
Hardware problems
Cheating on tests and assignments
Changed family members perceptions of the student role
Interference of home affairs with online learning
Being far away from the clinical context and its consequences
Theme 4: The passage of time and the desire to return to face education
Initial confusion
Normalization
Waiting to return to face education

### Theme 1: Novelty of e-learning method

#### Weakness of infrastructure

Most of the students explained that it took at least a month for the university’s Navid E-learning system to be launched after the closure of the university. Students identified weaknesses of the system infrastructure, including inconsistencies in the system’s messaging to students about class schedules, interference of professors’ classes with each other, students’ lack of competence to enter the system and technical difficulties (for example, students reported being logged out of the Navid system spontaneously and unable to re-enter the class).

" E- learning did not have the prepared context at the start and it took until after NOROUZ holiday to start the process of uploading the professors’ files. After that, when we had an online class, I tried a lot but could not enter the system. I called the person who was responsible for the university’s E-learning and he said he would solve the problem but no progress was achieved.".(Participant 3, a student in semester 6)

#### Students’ unfamiliarity

The students were unfamiliar with this educational system and there was an absence of tools for students about how to operate the e-learning system. Students experienced various problems in communicating with the new educational system, in downloading educational files, uploading professors’ assignments, sending questions to professors and receiving feedback from professors.

" At first, I did not know at all how to send my homework to the professor or how to write my questions so that the professor would understand and respond." (Participant 7, a student in semester 8)

### Theme 2: Advantages of e-learning

#### Flexible self-centered learning

Because the professors used predominately offline (asynchronous) learning modules with uploaded class materials, students experienced independence in setting their learning program during the E-learning so that they were able to access the content based on their desire to learn anytime and any where. The possibility of hearing the recordings of the instructors multiple times made it easier to write their notes and better understand the lesson. Each student used files based on individual learning methods such as listening to the recordings several times, writing notes on the recordings at the time of listening, watching the slides and then comparing their notes with the content of the reference books.

"Well, I downloaded and listened to the professors’ files whenever I had free time and I was not bored, and whenever I got tired, I would stop and listen again. I could manage to study, for example, as "I was sitting on the bus, listening to professor’s voices and I was comparing to the pamphlet." (Participant 5, a student in semester 6)

#### Reducing concerns with face education

Students had previously experienced some concerns with face education; these concerns were moderately reduced or eliminated with E-learning, including commuting tiredness, feeling sleepy caused by waking up early in the morning for classes, the stress of managing household chores, including babysitting, cooking, and other roles, the financial problems of living in a dormitory, and the cost of commuting to the university.

" It was good for us who stayed in the dormitory to be at home and not to commute, and we no longer had the stress of spending money and we were at home at that time when it was wasted on commuting and we could finally study our lessons in the right conditions." (Participant 7, a student in semester 6)

### Theme 3: Disadvantages of e-learning

#### Poor communication between professors and students

Implementing E-learning caused the students to lose the opportunity to communicate in person with professors. In some courses that used only offline learning methods, students did not know the professors at all and did not have a mental image of the professor’s face, teaching style, and moral and behavioral traits. Without direct contact with the professor, just hearing the professor’s voice, interfered with establishing effective communication between the student and the professor. Students reported that the professors only talked about the content, without other communication such as greeting or saying goodbye or giving an example to better understand the subject. They noted that the use of a uniform voice tone during teaching without observing the professor’s body language reduced the effectiveness of the communication. Furthermore, not receiving or waiting for feedback from a professor concerning their assignments and questions disrupted the communication process and left the student unwilling to send further messages to the professor.

Students had some opportunity to interact co-operatively with the professor and friends in courses with online learning. Virtual classrooms created the possibility to observe the professor’s face and voice simultaneously which enabled them to understand the lessons better because they could ask their questions and solve them with the help of the professor. However, frequent chatting, noise created by students, and the large number of questions asked of the teacher reduced the quality of communication and learning.

"Some of my professors were totally new and we had not observed them at all and I had no knowledge about professor’s shape and prestige and it was extremely significant to me what the professor’s face looked like".

"Some professors show some points with their hand gestures at the time of teaching that enables us to understand better, but E- learning is not the same and you cannot make a real connection with your professor." (Participant 2 a student in semester 6)

#### Decreasing the interactions among the classmates

Students’ communication was limited during E-learning courses and was restricted to coordination for homework and group work. Unlike face-to-face learning, students lost the opportunity to talk, have free time with each other, go to the dining hall and the prayer hall, and the ability to learn from each other.

"Corona situation caused us to miss contact with our friends and spend time together in the face learning where we visited each other. We were highly good together. We went to the dining hall and asked our problems in lessons and compared our pamphlets, but we were not able and had no opportunity to do this in E-learning." (Participant 9, a student in semester 8)

#### Problems with education files

Students frequently experienced various problems such as poor audio quality in education files, additional sounds during audio recording, unknown start and end of the audio, very short and brief slides, slides with a high volume of content, unfamiliar English terms on slides, a large number of slides, long audio and the wrong files.

"Some files had a problem and you observed that a professor talked for an hour and the sound was recorded so badly and we heard the extra sound of the professor’s house and there were 100 slides and they were all full of text and it was very difficult to turn them into a workbook." (Participant 6, a student in semester 4)

"We heard some terms for the first time and that term should have been written in the slide while the slides were too short and did not contain the terms completely. "For example, in the category of ventilator modes, the master says in the recorded voice that he used to pronounce cpap and the term completely and quickly, but it is not in the slide, and you have to listen to this new term several times and you will not understand it again." (Participant 8, a student in semester8)

#### Superficial learning

Students compared their learning in face-to-face format with E- learning, frequently declaring that their learning was less and more superficial than before, resulting in a lower grade point average. The students understood that they played a significant role in this superficial learning due to not focusing on the content, carelessness while listening to the professors, and not studying the course files in a timely manner. They reported collecting files for the end of the semester, studying the questions on the night of the exam, not studying the questions thoughtfully, not studying some parts of a lesson and not understanding some of the content.

Professors also played a role in superficial learning through lack of professor’s supervision of the learning process and not performing developmental evaluation during the semester to determine the level of learning; these factors deterred students from deep learning.

"E- learning was not like face-to-face learning and there was no opportunity to study the files carefully and once you noticed that the whole file was not studied and then you had to study carelessly and did not understand many things." (Participant 5, a student in semester 6)

"The professors contributed to poor learning and many of them did not evaluate anything, no questions, no solutions, no follow up, and some of them uploaded their files too late and there were no files for two weeks and sent all slides at once, "and well, we did not have time to study and we neglected it." (Participant 5, a student in semester 6)

#### Hardware problems

Students frequently experienced problems with power outages, internet interruptions, having no laptops, having mobile phones without advanced technology, and having no access to reference books.

" We did not have a laptop and we have a computer and I called the electricity office and said that my exams will be held in two weeks and our living area is somewhere and I asked you not to cut off our electricity but the electricity was cut off and My internet was not connected and I called the college tutor and followed up".(Participant 3, a student in semester 6)

#### Cheating on tests and assignments

Students reported cheating on e-learning exams and sharing homework in e-learning. Most students attempted to respond to the exam questions using the available notes and books, or by dividing the lesson materials among their classmates, with each person responsible for studying and understanding a section. Access to books and pamphlets and the possibility of searching the Internet led to students not to rely on their real knowledge during end-of-semester assessments or assignments. They helped each other respond to the test questions by searching the Internet and sharing information. Scores on tests and assignments may therefore not represent the real academic achievement level of the student.

"Well, there is a difference among students in face-to-face learning and e-learning, and that means the student shows him/herself to be more educated and at a higher level of science by using the booklets, and having access to quickly search and find the response to a question, It means that you do not rely only on your own knowledge." (Participant 4, a student in semester 4)

#### Changed family members perceptions of the student role

Family members’ expectations of the student were not primarily related to the role of the student and active participation in E-learning; they expected that students perform other roles that were assigned to them alongside other family members such as housekeeping, shopping for the house and accompanying them to social events.

"When you are at home, you should be like everyone else, and for example, if they want to go to the party, you say I have an online class and they say, ’Well, turn it on and listen, on the way, and you have to go with them and they do not look at you as a student in the E- learning." (Participant 5, a student in semester 6)

#### Interference of home affairs with online learning

The students explained that the time at which online classes were held interfered with household chores and responsibilitied. Some professors held online classes from 13:00 to 15:00, which were at the same time as prayers, lunch, and half-day breaks for family members. In other cases, family members were watching TV and talking to each other, and younger siblings were playing during online classes. These activities decreased the student’s focus on the content that was being presented.

"I was not able to concentrate on the content at home when I was in the online classroom and the class was held at the same time with my chores, I am washing the dishes and also listening to the voice and I did not have the accuracy that I have to have in the classroom and write a pamphlet and the conditions are not ready at home, hence, learning and accuracy are decreased." (Participant 4, a student in semester 4)

#### Being far away from the clinical context and its consequences

Nursing students reported that only theoretical courses were offered to them with the closure of the university and the internship units were not presented for a long time, causing them to experience weakness in communication with patients, forgetfulness and decreased self-confidence in performing clinical skills, and fear of developing weakness in professional skills.

"I no longer accept myself because I forgot the little thing that I learned in the internship course, and we even forgot the way to communicate with the patient and ask the history, which that we had just started and I planned to do better. We also left the rest of the tasks, such as giving medicine and taking blood vessels, and our practical work is extremely weak because we could not practice." (Participant 9, a student in semester 8)

### Theme 4: The passage of time and the desire to return to face education

#### Initial confusion

The students all agreed that they were confused and shocked at the start of E-learning. They questioned each other about the way to offer lessons, because they were unaware at the beginning of the university closure how their education would be conducted. They progressed with trial and error in using the E- learning system.

"First of all, most of the students were confused and did not know what to do, and we did not really understand what offline means, what online means, and what we should do." (Participant 10, a student in semester 4)

#### Normalization

Students gradually became familiar with the process of interaction with the E-learning system within a few weeks, and learned the way to continue the learning process in this type of education and to interact with professors in E-learning. They became familiar with the situation and processes.

"I understood everything after almost a month and I already knew how to get the files and how to ask my questions and send my assignments. Presently, we have had two semesters and we are completely used to working with it, and it has become currently normal for us." (Participant 4, a student in semester 4)

#### Waiting to return to face education

After months of e-learning and comparisons with the previous face education, the consequences, disadvantages and advantages of this education had resulted in most students preferring face education. They desired to return to the school environment, communicate with classmates, professors and patients, spend leisure time with classmates, and use the facilities of the school.

"I think it’s correct that e-learning has some goodness, but it’s not worth it, and it can’t replace the presence of training. I’m willing to go and get tired, but I’m going to class and I’m expecting to go back to college where I can take my unanswered questions and connect with my coaches and with my patients to strengthen my skills." (Participant 4, a student in semester 4)

## Discussion

This research described nursing students’ perceptions of e-learning during the Covid-19 pandemic. The e-learning educational method was a new approach with which both students and their professors were unfamiliar and for which infrastructure was initially inadequate. Over time as familiarity with the new learning method and resolution of some of the technical and infrastructure problems, students perceived both advantages and disadvantages of e-learning, with the conclusion that e-learning is complementary but not a substitute for face-to-face learning,

Students mentioned self-centered flexibility learning as a perceived advantage of E-learning. Other research have addressed similar benefits of e-learning such as continuous accessibility to educational contents [18–20], opportunity to learn based on personal speed [5, 18] and flexibility [5, 21, 22]. Another perceived benefit of e-learning in our study population was the reduction of the concerns arising from going to university, the associated expenses, and arranging home schedules with academic affairs. Similarly, previous studies have reported significant advantages of e-learning are saving students time due to the lack of need to move and travel to the place of study, being comfortable, and cost-savings (such as cost of cars, gasoline, book purchases, office and paper supplies, and food preparation) [5, 22]. However, also similar to the findings in our study, family and social roles complicated student participation in e-learning. Lack of suitable space at home for e-learning without noise from family activities and interruptions from family responsibilities have been reported as disadvantages of e-learning [5, 22, 23].

While advantages were recognized by the students, disadvantages dominated the conversations with our participants. These disadvantages centered around the technical problems with learning in the electronic environment and on the nature of the teacher-student interactive learning process.

Similar to results from Saudi Arabia [24], nursing students in our study pointed to the need for improved infrastructure and training of users to apply e-learning. Electronic access to e-learning was problematic for some students. Issues such as lack of required hardware, internet disconnection, and power outages were experienced. Students in rural environments or in families with multiple members in need of simultaneous internet access are particularly impacted [23]. Problems with the electronic files were frequently mentioned within the theme of e-learning disadvantages as technical issues that created dissatisfaction among our students. A previous study identified the need to correct defects in educational files such as loading audio files without slides, loading audio slides, large volumes of content, slides without adequate explanations, high-quality audio slides and slides with non-Persian language was the reason of students’ dissatisfaction with e-learning [25]. Hayat et al. in this regard noted that the presentation of content in e-learning must be based on standards for quality education and professors must be trained in the skills necessary for e- learning [26]. The availability of a technical supporting team is critical to successful development, deployment, and ongoing problem-solving of infrastructure issues [18].

A major source of dissatisfaction with e-learning was the decrease in opportunity for bilateral interaction with professors. Absence of face-to-face communication and lack of feedback from professors inhibited the formation of an effective teaching-learning relationship, which can create frustration, depression and reduced motivation in students [22]. Interaction with professors and timely feedback is essential to the success of the education process [24] and inadequacies in these areas are commonly reported by students participating in e-learning [22, 25–27]. In parallel with the change in students’ interactions with professors, communication, group learning activities, and supportive interactions with classmates were also reduced with the shift to e-learning. Students perceived that this isolation interfered with their learning [8].

Superficial learning was noted by all participants as a consequence of the change to e-learning during the pandemic to which both students and professors contributed. For example, lack of clarity of learning objectives, supervision, teacher-student interaction, and feedback to students are obstacle to the learning process [28, 29] and were among the primary causes of superficial learning [29]. Likewise, failure to be a self-motivated learner can lead to distraction, decreasing the student’s focus on content and assignments and attendance at online classes [22].Students reported more cheating than with traditional face-to-face instruction methods. In lieu of adequate preparation, students distributed the workload with other students, working together to access books and internet sources in answering test and assignment questions. Professors must adapt testing methods within e-learning to assure the validity and reliability of assessment results and to document each student’s individual learning achievement [26].

A strongly perceived disadvantage of closure of university classes and clinical experiences was the loss of clinical context. Students lost the possibility of applying and practicing the communication and technical skills they normally acquired in the clinical settings with their patients. Providing clinical context is important to the development of skilled nurses; this cannot be replaced entirely by E-learning. The use of simulation technologies can facilitate students’ learning in conjunction with virtual modalities [23]. In our setting, we were not able to operationalize alternatives to clinical experiences such as the use of virtual patients in simulated real scenarios [30], or standardized remote patients, where students have the opportunity to communicate with patients over internet to reflect a real clinical situation [31].

The passage of time improved the students experiences with the unplanned E-learning. Students first experienced confusion; over time normalization of E-learning occurred. However, they eagerly awaited the return of face education because they learn better in physical classes [32] They do not regard e-learning as an alternative to face education, consistent with prior studies with nursing students [23] and medical students [5], who did not regard this kind of education to be an alternative to clinical education in developing clinical competency.

### Study limitations

Only students in 4^th^, 6^th^ and 8^th^ semesters were included in the study. The experiences of early semester students were not assessed because they did not have the experience of transitioning from face-to-face to e-learning. Surveying the experiences of new students entering E-learning will likely produce equally important through different results. The researchers of this study were faculty members of the university and therefore their perceptions and assumptions about the transition to e-learning may have biased their interpretation of the data. The data were analyzed separately by each researcher and dialogue led to consensus on thematic categories.

## Conclusion

Students’ experiences indicated that e-learning was a novel method for their nursing education that temporarily replaced face education during the COVID-19 pandemic period. Students became familiar with this method over time as they experienced its advantages and disadvantages. They came to desire and expect the return of face education, recognizing that e-learning cannot replace face education in their minds. Because of the newness of this method of education and the lack of familiarity for students and professors and lack of strong and reliable infrastructures, it is incumbent on education managers and technical experts in e-learning systems to continuously assess and strengthen the technologic infrastructures and provide the necessary training courses for students and professors to empower them within this new learning modality. New types of hybrid education have been accelerated by the COVID pandemic crisis and are likely to remain the mainstream educational model. In particular, professors must learn to produce high quality content using the newest audiovisual presentation methods, and establish mechanisms for effective student-professor interactions, feedback, and periodic evaluations to promote deeper, non-superficial learning. Developing strategies for integrated virtual simulations to support clinical learning will be needed to bridge the gap between theory and clinical practice in order to enhance the quality of education. Further studies by stakeholders of these new educational formats, including professors and specialists of hardware and software, will be helpful for enhancing e-learning systems.
